# Re-engineer apparel manufacturing processes with 3D weaving technology for efficient single-step garment production

**DOI:** 10.1016/j.isci.2024.110315

**Published:** 2024-06-22

**Authors:** Yuyuan Shi, Lindsey Waterton Taylor, Amanda Kulessa, Vien Cheung, Abu Sadat Muhammad Sayem

**Affiliations:** 1 Digital Textile Lab, School of Design, Northumbria University, Newcastle Upon Tyne NE1 8ST, UK; 2 3D Weaving Innovation Centre, School of Design, University of Leeds, Leeds LS2 9JT, UK; 3 Institut für Textiltechnik, RWTH Aachen University, 52074 Aachen, Germany; 4 Manchester Fashion Institute, Manchester Metropolitan University, Manchester M15 6BJ, UK

**Keywords:** Applied sciences, Engineering

## Abstract

Traditional apparel assembly technology—cut and sewn process—requires labor-intensive pre- and post-production. While conventional weaving technology has made efforts to streamline the garment-making process, additional assembly processes are still required—sewing or joining after removing the woven samples from the loom. This challenge in the garment-making process discloses the need for a novel type of advanced textile technology and manufacturing techniques incorporating shaping and assembly capabilities. Exploiting three-dimensional (3D)-to-two-dimensional (2D)-to-3D methodology integrated 3D weaving technology, the 3D woven bra prototype is practically demonstrated in a significantly effective manufacturing process, shaped in one weaving cycle without additional assembly needs. The bra manufacturing process is also assessed by traditional industry loom, and the same efficient manufacturing process is also achieved. This indicates that 3D weaving technology contributes as an innovative manufacturing technology in the apparel industry to facilitate the manufacturing process significantly and eliminates further joining and sewing processes.

## Highlights

• 3D weaving technology is innovatively applied as garment-making technology
• Eliminates the need for additional sewing or joining processes
• Produces complete 3D woven apparel in a single manufacturing step
• Integrates 3D-to-2D geometric and 2D-to-3D manufacturing processes

## Graphical abstract

**Figure:**
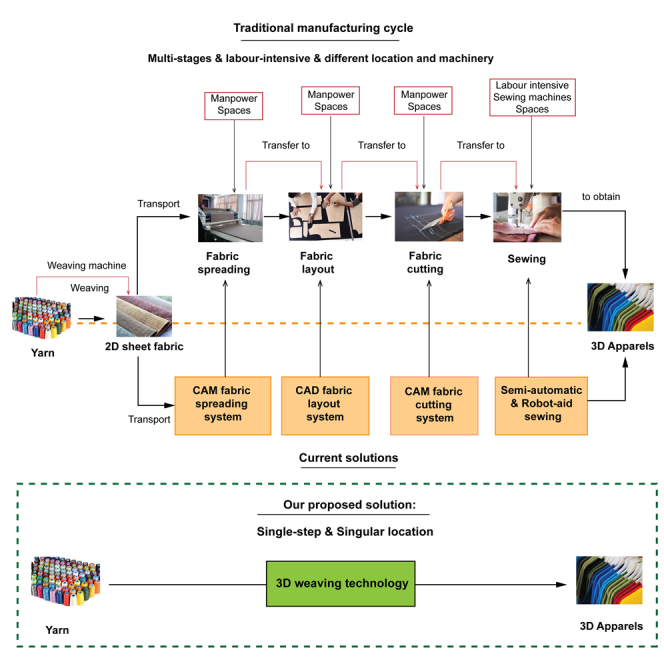

## Introduction

Cut-and-sew technology is the primary technique for making two-dimensional (2D) sheet fabrics into three-dimensional (3D) shell clothing forms.^1^ Sewing is the most common joining technology applied in the apparel industry^2^^,^^3^^,^^4^ to stitch two or more plies of fabric together using needles and threads. The manual sewing process, which is labour-intensive,^5^^,^^6^ is still the norm in the apparel industry, as it has a low manual cost and flexibility of replacing blocks.^7^ However, the disadvantages of sewing are obvious. The cut-and-sew technique based on one ready fabric resulted in up to 40% of waste.^4^

The integration of weaving technology into the garment-making process has led to the emergence of fully fashioned woven garments. Each garment piece, formed by garment block, was woven individually. After removing from the loom, these garment blocks were cut and then sewing them together transfer to 3D garment forms. A few practice-led research has been explored in fully fashioned weaving garments to reduce material waste. One prominent method involves optimizing the layout of garment patterns to reduce waste, as explored by Mcquillan, 2020. 3D digital software—CLO3D—was employed as digital tool to reduce the material waste in the pattern construction trial-and-error process.^8^ 2D stocked T-shirt pieces are woven, then cut, and separated from each other after removing from the loom. The final T-shirt was sewn together. This method enables to T-shirt to be constructed conventionally without spreading process, but the sewing time did not reduce. Piper and Townsend (2015) developed the woven garment that can be worn after being cut from the loom.^9^ It is claimed that it streamlines the production process simultaneously. This innovative technique enables minimal material consumption and cutting waste. However, the use of a hand dobby loom presents limitations due to the harness lifting mechanism, resulting in oversized garments with a straight shape and simplistic silhouette. Similarly, Lefferts (2016) also explored the fully fashioned woven garment; the 2D garment blocks were woven using synthetic yarns. Subsequently, the woven 2D garment pieces were removed from the loom and joined together using heating joining technology after being cut.

The previous works have made efforts to shorten garment making process, but additional assembly processes—sewing or joining—are still required after removing wovens from the loom. All these call attention to a need for an alternative textile technology for the sustainable and efficient apparel manufacturing process formed by anthropometric database. 3D weaving technique has been introduced into broad engineering industries due to lighter characteristics and customized tailored end applications. Previous studies on 3D weaving have highlighted works on 3D shell fabrics, 3D woven nodal truss structures,^10^^,^^11^^,^^12^ and 3D dome shapes^13^^,^^14^^,^^15^^,^^16^ for technical and engineering industries. 3D weaving using advanced weaving technology is the development of a composite form that may contain hybrid fibre-yarn blends and multiple layers (warp) and levels (weft) to interlock.^17^ The latest 3D weaving technology with the interlocking of the yarns in the x (longitudinal-warp), y (cross-weft), and z (vertical-through-thickness) directions, alongside the tailored location of amalgamated weave architectures. This approach, which utilises multilayer (warp) and multilevel (weft) woven structures, enhances specific drape, support, and movement characteristics.

In this research, 3D weaving technology is innovatively applied as garment-making technology via 3D-to-2D-to-3D process. This process is divided into two phases: 3D-to-2D geometric process and 2D-to-3D manufacturing process. 3D-to-2D geometric process is to flatten 3D scanning geometry into 2D geometry; the aim of geometric process is to obtain the 2D flattened (2DF) geometry of bra with defined areas for weaving parameter arrangement and shape reference. 2D geometries with appropriate weave architectures transmitted/unfold back to 3D woven apparel forms are identified as 2D-to-3D manufacturing process. The 3D-to-2D-to-3D research process by exploiting the innovative 3D weaving parameters aims to seamlessly transform yarns into complex 3D garment forms within a single weaving cycle. This eliminates the need for additional assembly processes, streamlining the manufacturing workflow.

## Results

### Bra prototype results

**Figure 1:**
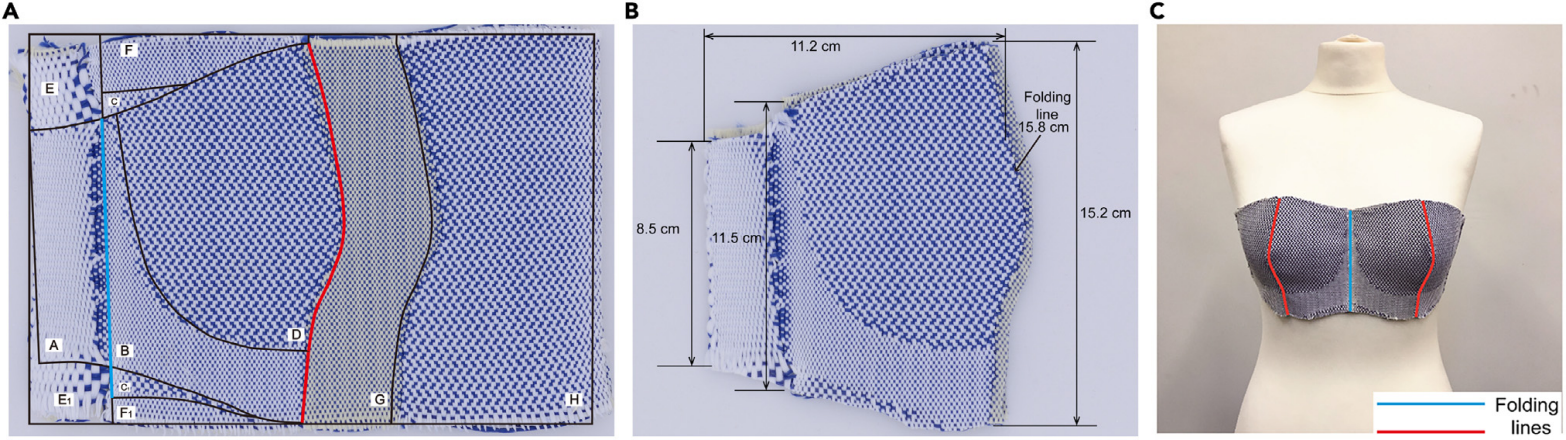
3D woven bra prototype results (A) The folded bra prototype taking off loom. (B) The flattened 2D form of bra. (C) The final 3D woven bra front block.

MS-100 loom was applied to weave this bra prototype, with provided 167dtex polyester. The pre-trails were also applied to test the weaving density. The pre-trial was conducted to adjust the actual weaving density to ensure that the final bra meets the required geometric size design. Initially, pre-trial testing with a design weaving density of 24 wefts/cm resulted in a smaller size than required. As detailed in the weaving density calculation section of the Methods, the final weaving density was calculated and adjusted to 48 wefts/cm to produce the final bra prototype meeting the size requirements. The final bra front block and measuring data are shown in Figure 1, the red and blue lines are folding lines. MS-100 loom is the latest weaving technology and it can be regarded as the prototype weaving loom. This 3D woven bra sample was woven completely in one weaving cycle. It is a much more streamlined manufacturing process comparing with the existing bra manufacturing technologies. The evaluation of the shape of the woven bra is to compare the 2D folded woven sample shape with the corresponding 2DF geometry. The underlying principle is that the 2D folded woven sample should exhibit identical shape and size characteristics to the 2DF geometry. The findings of woven results revealed a slight difference between the size of the 2D folded bra and the original 2DF geometry. This deviation can be attributed to the natural shrinkage that occurs after the removal of the sample from the loom without tension hold.

### Sample results with the traditional loom

**Figure 2:**
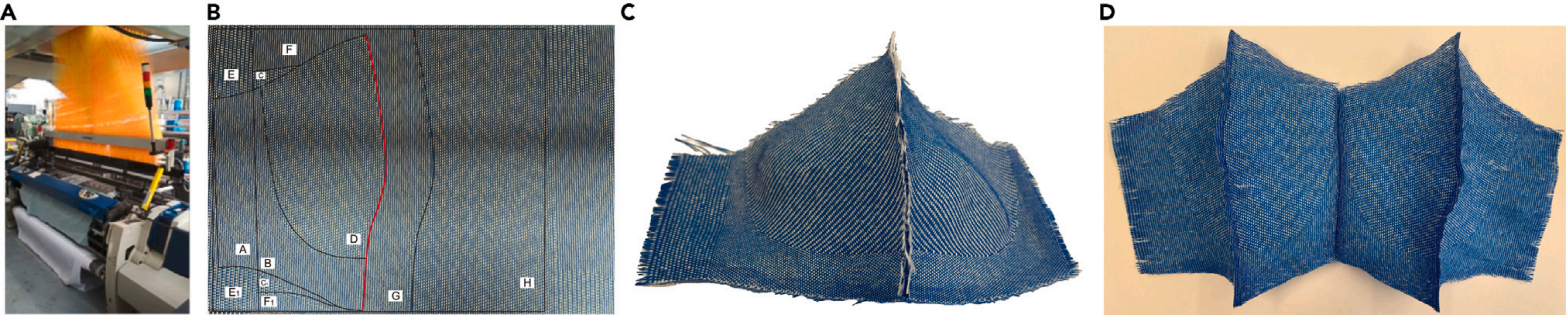
Jacquard rapier loom woven bra results (A) Picanol Optimax rapier loom with Bonas Jacquard. (B) 2D form with segments. (C) Two-layer woven sample using rapier loom. (D) Four-layer weaving sample using rapier loom.

Jacquard harness rapier loom is the mainstream machine occupied by most weaving companies. The advantages of rapier weft insertion are well known, such as highly speed and simple weaving parameters. However, seams and cutting-off are not avoided due to the continuous wefts and shuttleless weft insertion. Picanol rapier loom with Bonas Jacquard harness (date of production: 2007), as shown in Figure 2A, was utilized to test the feasibility of 3D weaving bra designs using a traditional Jacquard loom. The Picanol loom configuration parameters are shown in Table 1. The available weaving width of Picanol rapier loom is 190 cm, which enables weaving at least nine bra samples simultaneously. The 2DF bra with segments for the rapier loom is displayed in Figure 2B. 2L and 4L bra samples have been woven successfully, as shown in Figures 2C and 2D. The density of 4L bra sample is very loose because only one warp beam is available in the back of this traditional loom. The success of bra manufacturing endeavors has validated the theoretical 3D weaving design of the bra samples. This demonstration illustrates that the theoretical design of the bra samples can not only be realized in advanced Jacquard loom (MS-100 loom) but also on industry-standard traditional looms.

**Table 1:** Picanol rapier loom configuration parameters

Picanol Loom	Shedding system	Jacquard single-end control
Warp setting	Weft insertion type	Rapier
	Yarn	2/36 NE Polyester
	Width	190 cm
	End number	2368
	Reed	Flat-Reed
	Beam number	1
Weft setting	Yarn	2/36 NE Polyester

## Discussion

The application of the 3D-to-2D-to-3D methods, combined with optimized 3D weaving parameters, has significantly streamlined the bra manufacturing process, eliminating the need for additional sewing processes in our research. The successful development of a 3D woven bra prototype, alongside a prototype created using traditional industrial looms, underscores the feasibility of the developed 3D weaving principles in bra manufacturing. A notable achievement is the creation of the 3D woven bra prototype, which seamlessly unfolds back into its 3D apparel form after being removed from the loom without requiring any supplementary sewing or joining procedures. This demonstrates that bra shaping occurs within a single weaving cycle, directly transforming yarn into 3D woven apparel forms. Moreover, the utilization of traditional looms to test and validate this process suggests the potential for the aforementioned bra prototype design to be integrated into future industry production, offering highly streamlined manufacturing procedures.

The proposed 3D-to-2D-to-3D methodology has been developed to effectively shape yarns into 3D woven bras, streamlining production by eliminating the need for additional sewing steps. This approach was chosen as the primary research method due to its alignment with both 3D weaving and apparel manufacturing processes. Its efficacy has been demonstrated in previous work producing 3D woven nodal structures^10^^,^^11^ enabling the creation of lightweight composite truss structures. In this process, the 2D form transitions into 3D woven structures upon removal from the loom, obviating the need for additional joining processes. Moreover, the 3D-to-2D-to-3D method aligns with traditional garment-making processes. Initially, the 3D body surface is translated into 2D garment patterns/pieces, which are then assembled through sewing before being transformed back into 3D garment forms. In our research, the 3D-to-2D garment geometric process involves flattening the bust area of scanned 3D digital models into 2DF garment geometry with the assistance of 3D CAD/CAM software. Multi-layer and multi-level weaving architectures are subsequently designed to produce the 3D woven bra prototype. The efficacy of this weaving design is assessed through actual loom settings, leading to the production of the final 3D woven bra results. Utilizing 2DF geometry derived from scanning data ensures the precise size and geometry necessary for the weaving process, while 3D weaving parameters are meticulously designed to shape yarns into 3D forms. This ensures that the resulting 3D woven bra samples adhere to the proposed design and are shaped accordingly during the weaving process.

**Figure 3:**
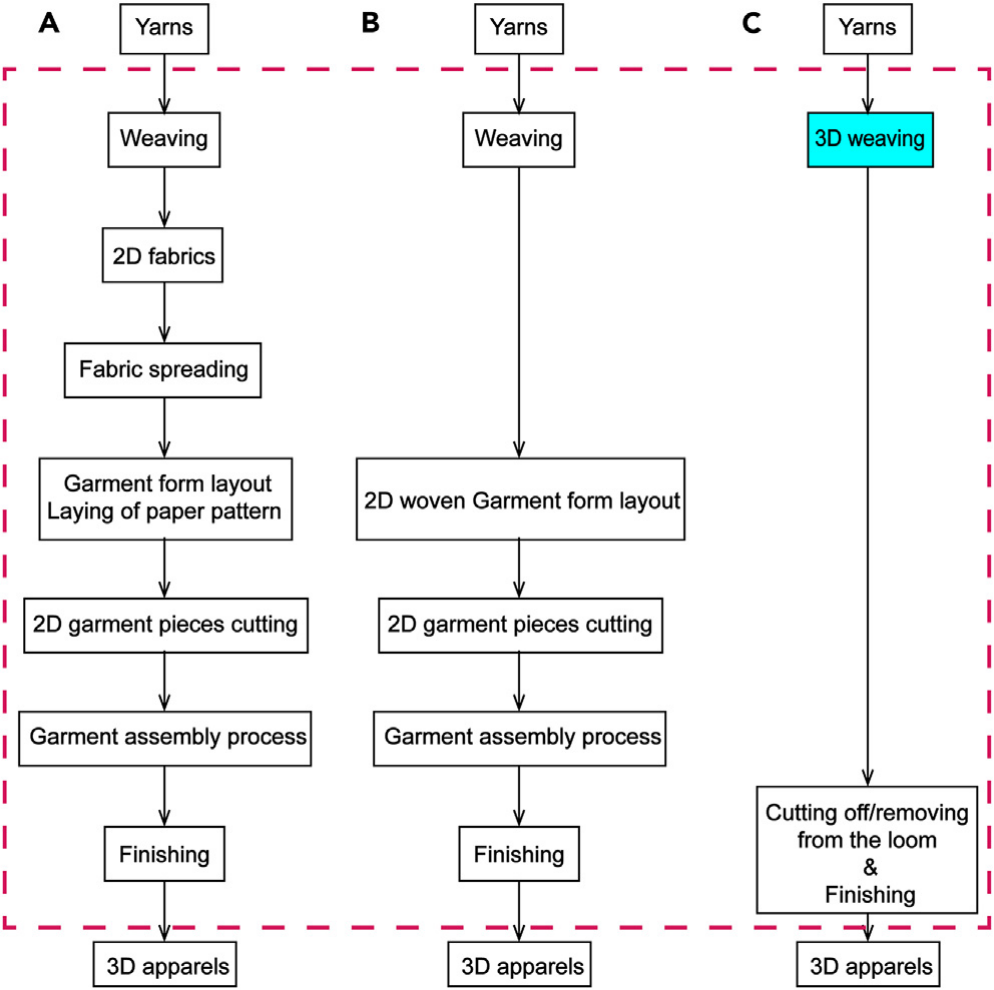
Comparisons of yarn to 3D apparel making processes (A) Traditional apparel making process-weaving fabric. (B) Fully fashioned woven apparel making process. (C) 3D weaving apparel making process.

The conventional method of apparel and bra production follows a cut-and-sewn process, commonly known as the 2D-to-3D process. In this approach, 2D fabric pieces are manipulated through manufacturing steps to achieve 3D shell forms that conform to the contours of the human body.^18^ Sewing technology has long been the primary method for assembling bras and apparel in the industry, involving the stitching together of fabric pieces and clothing blocks using needles and threads. While widely used, this traditional assembly technique has significant drawbacks. It often results in high material waste^4^^,^^19^ and requires extensive pre- and post-production processes.^20^ The labor-intensive nature of this manufacturing process can contribute to up to 40% of waste, encompassing material waste as well as resources such as human labor, utilities, and transportation.^4^ The cut-and-sewn from yarn to 3D apparel process is conducted in six steps,^1^^,^^3^^,^^21^ as shown in Figure 3A, which are: (1) yarn weave into 2D sheet fabric; (2) 2D sheet fabric spreading; (3) laying out of 2D garment block; (4) 2D garment piece cutting; (5) 2D garment piece sewing; and (6) garment finishing to obtain the finished 3D garments. Every step of conventional bra manufacturing exists in silos and requires their outputs to be transported to the next step. Different manual operations are required for each manufacturing stage, along with distinct machinery, with different skilled operators, before the material is transported to the following stages. Current research under Industry 4.0 have made efforts to improve efficiency and reduce labor required at each stage by exploiting a combination of CAD/CAM fabric cutting system, computerized semi-automatic system sewing machines, and advanced hierarchical robot-aided sewing.^22^^,^^23^^,^^24^^,^^25^ However, even with these advances, the nature of essential manufacturing routes remains unchanged. Each step remains isolated in distinct locations, necessitating transport with varying manpower involvement and machinery for different stages. These fundamental flaws with the conventional cut-and-sewn manufacturing process underscore the necessity for alternative manufacturing technology to re-engineer the bra manufacturing process. The existing apparel manufacturing process relies on transitioning yarns into 2D fabrics and subsequently forming 2D fabrics into 3D apparel through stitching techniques. In this research, we propose to shape yarns into 3D apparel within a single-step by exploiting 3D weaving technology as an alternative apparel-making technique.

Efforts have been made to use fully fashioned weaving garments to obtain a shorter garment making process,^26^^,^^27^ as shown in Figure 3B. However, this technology only eliminates the fabric spreading and layout process and does not reduce the time of garment assembly time. The significant benefits of 3D weaving technology are to eliminate spreading/plying, cutting, and stitching processes. 3D weaving technology can create 3D forms with complex near-net-shape geometries in a single weaving cycle, utilizing an independent weaving machine.^28^ The shape ability of 3D weaving principles and technology enable interlocking of yarns in the 3D directions, alongside the tailored locations of amalgamated multilayer multilevel weaving architectures. 3D woven near-net-shape forms have been successfully applied to automotive and civil engineering industry to eliminate addition joining processes and bonding requirements.^29^^,^^30^^,^^31^ The application and shaping ability of 3D weaving technology with the innovation of 3D weaving principles and technology is applied as apparel-making techniques, thereby enhancing the manufacturing process of apparel products.

The unique achievement of this research is to address the limitations of traditional garment-making techniques by innovating 3D weaving principles and technology. It offers an effective alternative that streamlines the manufacturing process—shape yarns into 3D apparel within one weaving cycle, as shown in Figure 3C, eliminating additional sewing and joining steps. The innovative application of 3D weaving technology in bra production fills a research gap and promotes effectiveness and sustainability in the apparel-garment manufacturing industry. These contributions drive significant advancements in both 3D weaving technology and the apparel manufacturing process, benefiting both areas and leading to more efficient and sustainable practices.

### Limitations of the study

One limitation of this research is that it focuses on producing an initial bra prototype centered around bra cup areas. The primary objective of this study is to establish a proof of concept, introducing 3D weaving technology as a garment-making technique and demonstrating its feasibility. Future research is recommended to explore the comprehensive manufacturing of complete bras using 3D weaving technology. This study is restricted to the production of a bra prototype using 3D weaving technology and does not encompass various types of apparel manufacturing processes. Therefore, further investigation is suggested to explore the application of 3D weaving technology in different kinds of apparel production.

This research developed 3D weaving principles and technology as garment-making technology and successfully applied it in bra prototype manufacturing. Based on the results and findings of this research, the following further works are recommended. 3D weaving technology has been developed to eliminate the sewing and joining process in bra manufacturing. To further expand its potential impacts in the apparel market, future works are recommended to conduct property testing to assess 3D woven bras, for instance, thermal comfort, tensile strength, impact testing, and flexural testing. Additionally, it is advisable for future research to conduct practical testing by inviting individuals to try on the bra prototypes. This process will yield invaluable insights into their overall wearability and factors influencing consumer acceptance and adoption. The feedback gathered from these tests and individual try-ons will be essential for continually refining and advancing 3D weaving technology in garment manufacturing.

## STAR★Methods

### Resource availability

#### Lead contact

Further information and requests for resources and reagents should be directed to and will be fulfilled by the lead contact, Yuyuan Shi (yuyuan.shi@northumbria.ac.uk).

#### Materials availability

This study did not generate new unique reagents or materials.

#### Data and code availability

• This study did not generate statistical data.
• This paper does not report original code.
• Any additional information required in this paper is available from the lead contact upon request.

### Experimental model and study participant details

• This study did not involve an experimental model.
• This paper did not include any research with participants.

### Method details

#### Weaving loom and yarn application

Mageba multishuttle weaving machine with a Staubli UNIVAL 100 jacquard harness (MS-100) loom houses the single-end control Jacquard harness and four electric shuttles. The actual loom setting is shown in the table below. There are six beams in the back, enabling to manufacture of multi-layer and multi-level 3D weaving composites. Polyester (167dtex) is selected as the weaving yarns of picks and ends, which is most widely used in the sportswear and apparel industry, due to its durability, anti-wrinkle, and anti-stretch properties.^32^

**Table:** MS-100 loom machine configuration parameters

MS-100 Loom	Shedding system	Jacquard single-end control	
	Weft insertion type	Electrical shuttles	
	Shuttle numbers	4	
	Shuttle boxes	Left 4	Right 4
Warp setting	Yarn	167dtex Polyester	
	Width	21 cm	
	End number	1024	
	Reed	V-Reed	
	Beam number	6	
Weft setting	Yarn	167dtex Polyester	

#### Weaving density calculation

To make the final woven sample size meet the predetermined design, the pre-trial weaving was required to be tested in advance. As a result, the final weaving sample dimensions were computed by Df=DdDdDpt. Where $D_{f}$ is the final weaving sample dimension, $D_{d}$ and $D_{pt}$ are design dimensions and the measured dimension of the pre-trial weaving test, respectively.

Similarly, the final weaving density was obtained by WDf=WDdWDdWDpt. Where $WD_{f}$ is the final sample weaving density, ${WD}_{d}$ and ${WD}_{pt}$ are design weaving density and measured weaving density of the pre-trial test, respectively. Notably, if the computed weaving density is too loose or too dense to weave, the pre-trial weaving test and the calculation process should be repeated after re-designing ${WD}_{d}$.

#### 3D-to-2D-to-3D manufacturing process

The 3D-to-2D-to-3D manufacturing process – 3D-to-2D geometric process and 2D-to-3D manufacturing process – of bra prototype was illustrated in Figure 4. The initial phase, the 3D-to-2D geometric process, is to convert the 3D scanned anthropometric geometry of digital mannequins into 2D flattened (2DF) garment blocks with segmentations and designed boundary lines. 2DF garment blocks define the weaving architecture arrangement areas and provide references for bra shapes and sizes. During this stage, the 3D anthropometric geometry of scanned digital mannequins is transformed into 2DF geometry with segmentations and designed boundary lines (3D-to-2D). The cross-platform 2D/3D CAD/CAM systems employed in the process ensure the obtaining of an accurate 3D and 2D parametric geometry database. Measurement of lingerie mannequins by the 3D human scanner and 3D reverse engineering software assist in establishing 2DF garment geometries. This process prepares the 2DF garment geometries for the next digital design and programming for technical weaving routes. The subsequent research phase is 2DF garment blocks with proposed appropriate weave architectures are tested on the loom, in order to obtain 3D bra samples after cutting off the loom (2D-to-3D manufacturing process) within a single weaving cycle. In this stage, the 2DF garment blocks, converted via the 3D-to-2D geometric process, were additionally constructed in BOK CAD. Multi-layer multi-level weaving architectures are designed and gathered in EAT 3D software to transfer the proposed design into a loom-readable file. The Jacquard loom was applied to test the above 3D weaving design and to produce the final 3D woven bra results.

**Figure:**
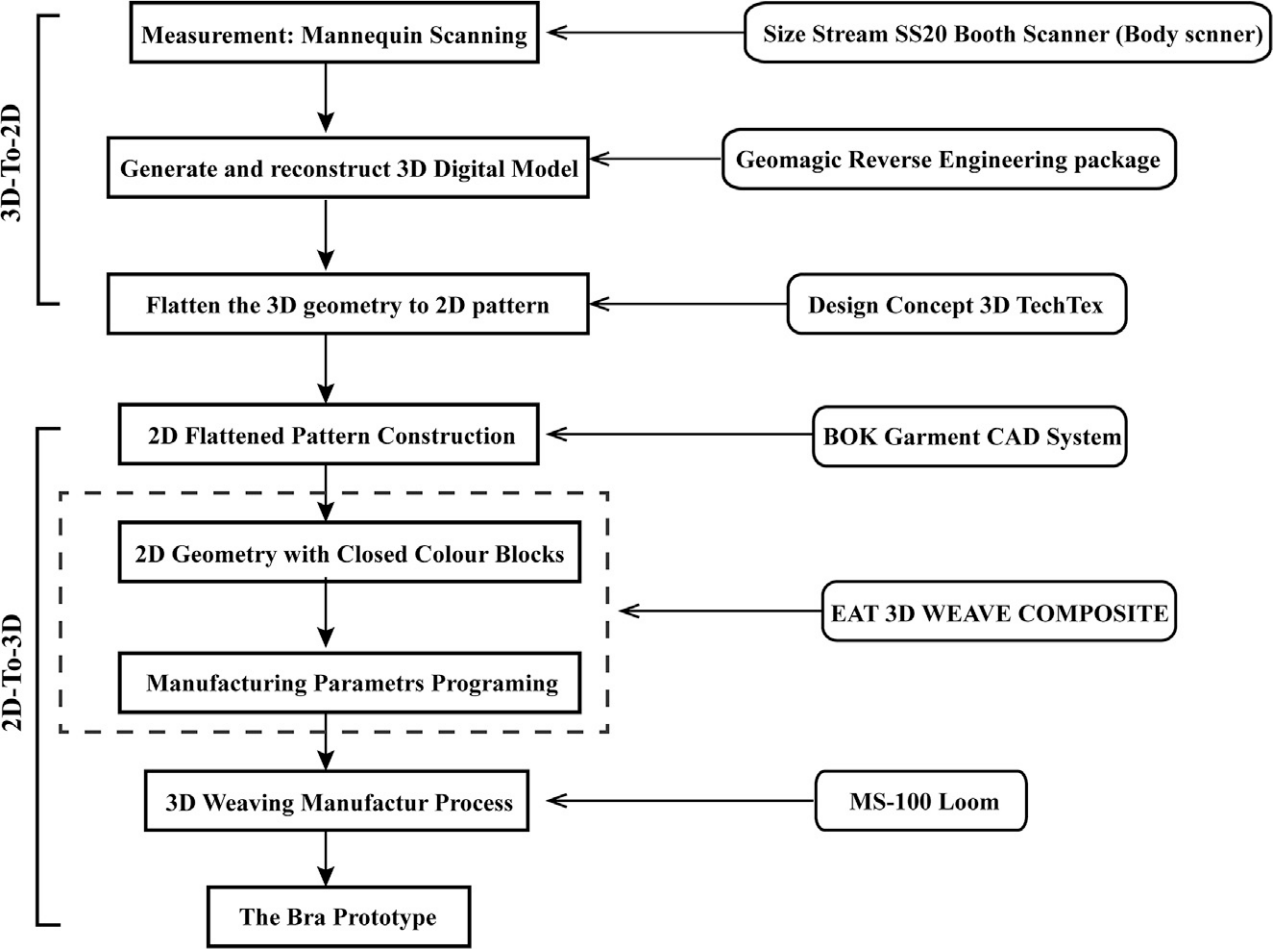
The 3D-to-2D-to-3D research process workflow

The cross-platform 2D/3D CAD/CAM systems employed in the process. The 3D surface geometry of the SS female sample (Size Stream, LLC) was captured by Size Stream SS20 Booth Scanner (Size Stream, LLC., SS20) and processed with a reverse engineering system - Germanic Warp (3D Systems, Inc., GW) initially in.obj data format. It was further processed in GW to reconstruct and repair the 3D geometry of the bust shape. The 2D garment blocks were flattened utilizing DesignConcept 3D TechTex (Lectra, DC 3D), based on the pre-processed 3D digital mannequin. This converted 2DF geometry was additionally inputted in BOK CAD with marked reference points. Finally, EAT 3D was applied to draw the 2DF geometry filled with multilayer and multilevel weaving architectures feed into latest MS-100 weaving loom.

The high-performance booth scanner SS20 (Size Stream, LLC) with automatic and 20 safe infrared depth sensors (available in dimensions: 80 inches (Height), 43 inches (Width), and 57 inches (Depth)) was exploited in this research. The scanned data were obtained as a.obj format file. This 3D digital data (.obj file) was imported into the GW (3D Systems, Inc.) to generate the digital model. Using 3D scanned digital models building accurate 3D anthropometric geometry reference data for flattening and for further 3D woven bra manufacturing. 3D scanning technology captures detailed measurements of the human body, ensuring an accurate representation of the complicated bust area. GW is a 3D digital model analysis software with advanced surfacing tools (3D Systems, Inc.). The polygon meshes firstly examined the scanning model in the reverse-engineering process. The “Mesh Doctor” function initially corrected the faulty mesh data automatically, and the utilization of “Polygons & Curvature” precisely repaired the 3D model.

#### Establishment of bra geometric transmitted process

DC 3D is designed for 3D/2D parametrically CAD for technical textile and material engineering and is more flexible with the shape or posture of scanning data. There are four modules with different file formats in DC 3D: 3D Design (for modeling).top, 2D Pattern (for flattening).pat, 2D Product (for acquiring 2D geometry).pro and 2D Draft (assembly/sewing plans).dft. The first three operations were primarily used in this research. 3D Design Module is used for 3D bra geometry design and drawing. The original 3D model file contains surface triangles, which is not necessary for the following steps. Rendering version was changed to Shading and Display edges to show a clear digital surface. Create region curves – Draw tool was utilized to draw the 3D geometry edges and curves on 3D digital model. The Move, Replace, Extract, and Insert tools were used to modify the reference points on curves. 3D regions were created by the Create regions – From Curves tool, based on surface curves for flattening. All constructed 3D regions are shown in Figure 5. In order to better match the edges of flattened 2D geometry, the Create notch target function was used to generate notches, and all notches were also shown in flattened 2D geometry. The new software module - 2D Pattern (.pat), was created for flattened 2D geometry. The Create Patterns – From regions function was exploited to select and flatten 3D regions to 2D geometry. During the flattening process, the flattening parameter was defined as Match edge lengths, as shown in Figure 5A. The final 3D and 2D geometry are indicated in Figure 5B. 2D Product (.pro) was created to save flattened geometry as a DXF file and then imported into BOK CAD for further operations.

**Figure:**
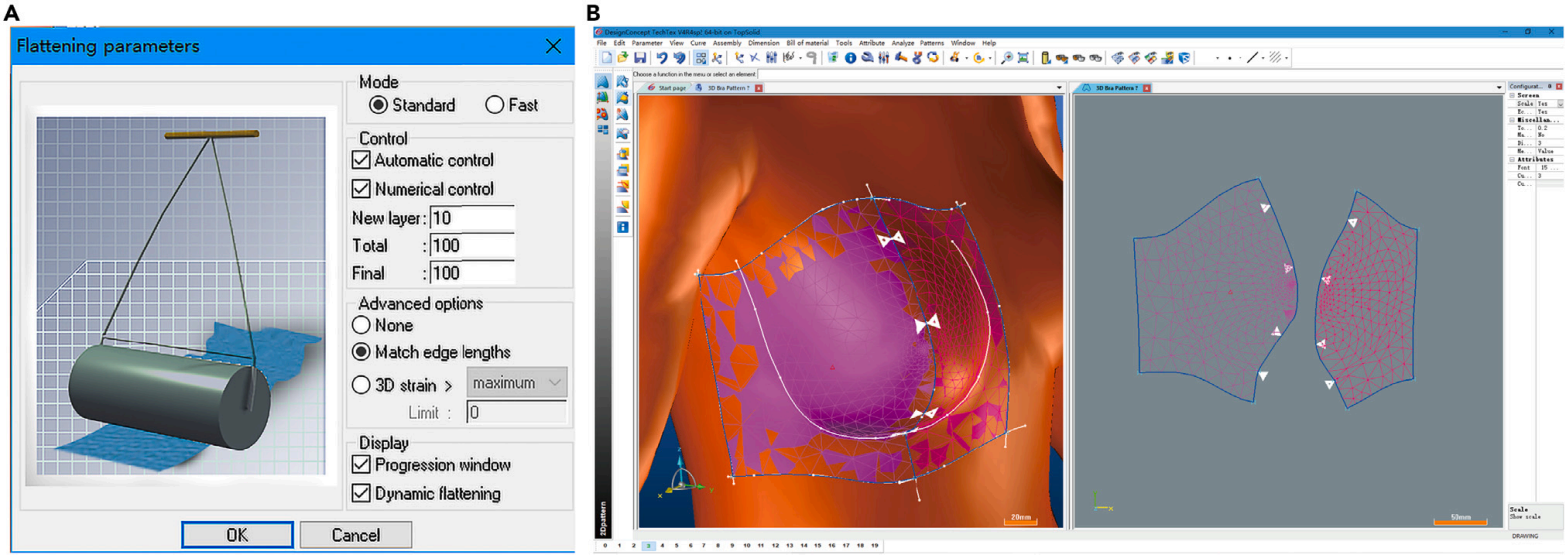
Flattening tool in DC 3D (A) Flattening parameters in DC 3D. (B) The screenshot of 3D geometry and flattened 2D geometry in DC 3D.

#### 2D bra geometry construction in 2D clothing CAD and textile CAD/CAM system

2D bra geometries obtained from DC 3D, was saved as DXF files and subsequently imported into BOK CAD for the parametrically drawing and then transferred to EAT software. After getting the drawings of geomatics conversational results with boundary lines and reference points in BOK CAD, EAT 3D was used to draw closed color blocks based on the calculated boundary line. Subsequently, color blocks were filled with corresponding multilayer multilevel weaving architectures. Figure 6A shows 2DF geometry of Bra prototype in BOK software. The measuring data of 2DF geometry of Bra prototype is shown in Figure 6B. The next step in EAT 3D is to fill colors into blocks, each color is assigned to one weaving structure, so the color areas were designed consistent with the arrangement area of the woven structure. The transferred 2DF geometry of Bra prototype in EAT3D is shown in Figure 6C.

**Figure:**
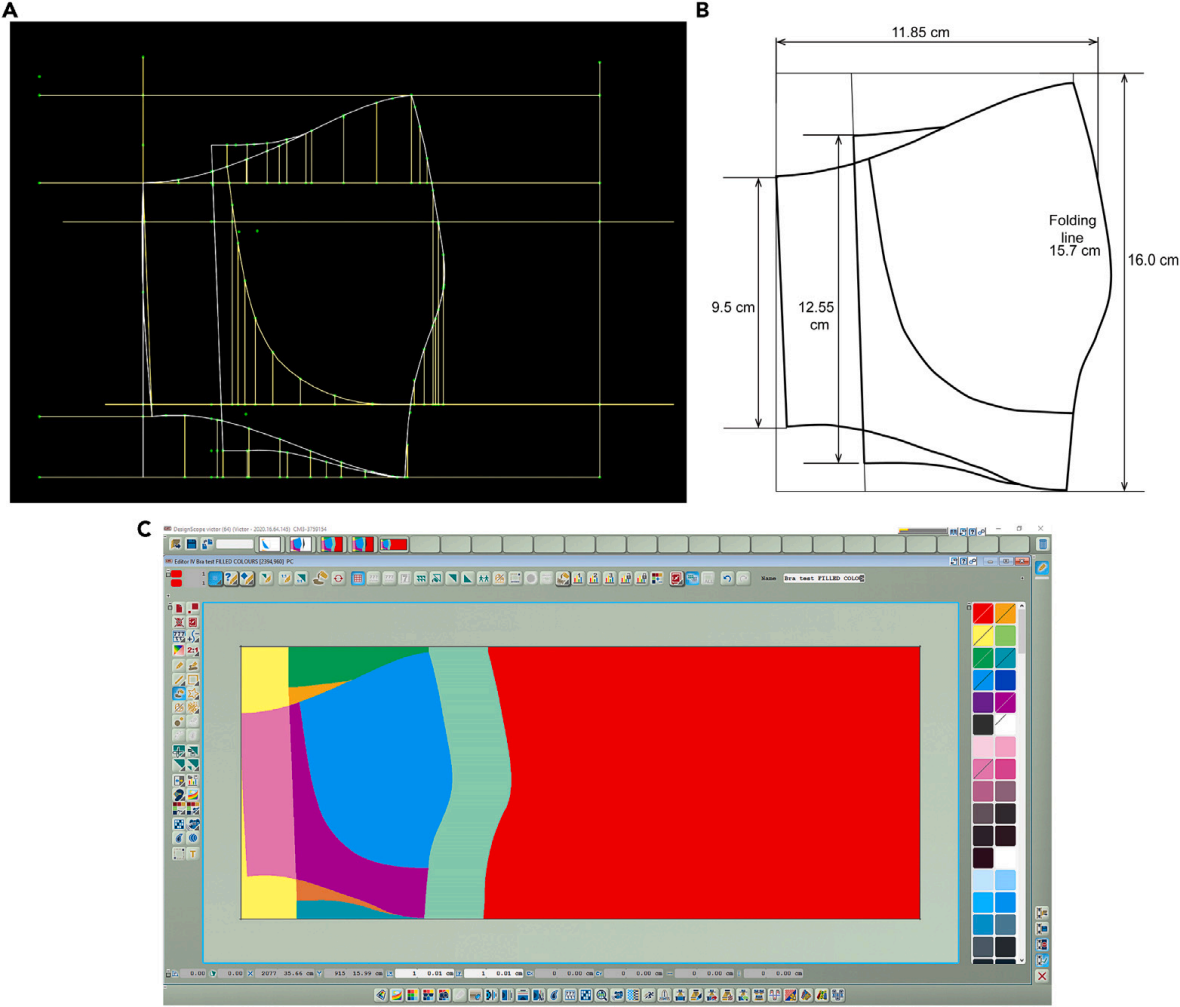
Drawing DC3D bra blocks in BOK and EAT 3D software (A) The 2DF geometry obtained from DC 3D drawn in BOK CAD. (B) Measuring data of 2DF geometry bra. (C) 2D geometry transfer to EAT software.

#### Weaving parameter design for bra prototype

Weaving parameters for bra prototype are presented in Figure 7. Divided segments of bra prototype are shown in Figure 7A. Segments named by different letters and different colors in 2DF of bra prototype were arranged with various multi-layer and multi-level weaving architectures. Segment A was arranged with 2L double faced 2/2 twill, Segments B and C were arranged with four-layer (4L) plain and 4L 2/2 Twill, respectively. The 4L Hopsack weaving architecture was assigned to Segment D and D_1_. The cross-sections of arranged weaving architectures were displayed in Figures 7B and 7C. The bra segments were arranged with various weaving architectures as shown in the table below.

**Figure:**
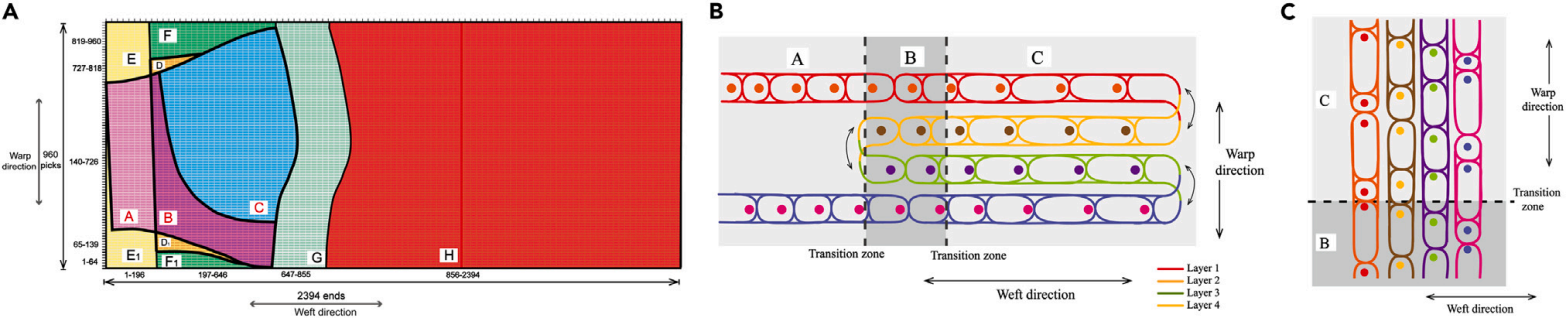
Bra weaving parameters (A) 2D geometric template of bra prototype. (B) The cross-section of warp direction of bra prototype. (C) The cross-section of weft direction bra prototype.

**Table:** The weaving architectures arrangements in different segments in 3D woven bra prototype

Segments	Weaving architectures	Segments	Weaving architectures
A	2L DF 2/2 twill	E, E1	2L hopsack
B	4L plain	F, F1	4L plain
C	4L 2/2 twill	G	4L plain interchanging
D, D1	4L hopsack	H	4L 2/2 twill

### Quantification and statistical analysis

This study did not use quantification or statistical analysis.
